# Alternative splicing regulation appears to play a crucial role in grape berry development and is also potentially involved in adaptation responses to the environment

**DOI:** 10.1186/s12870-021-03266-1

**Published:** 2021-10-25

**Authors:** Pascale Maillot, Amandine Velt, Camille Rustenholz, Gisèle Butterlin, Didier Merdinoglu, Eric Duchêne

**Affiliations:** 1 SVQV, INRAE - University of Strasbourg, 68000 Colmar, France; 2 University of Haute Alsace, 68000 Mulhouse, France

**Keywords:** Alternative splicing regulation, Grapevine, Fruit development, Abiotic stress, Adaptive traits

## Abstract

**Background:**

Alternative splicing (AS) produces transcript variants playing potential roles in proteome diversification and gene expression regulation. AS modulation is thus essential to respond to developmental and environmental stimuli. In grapevine, a better understanding of berry development is crucial for implementing breeding and viticultural strategies allowing adaptation to climate changes. Although profound changes in gene transcription have been shown to occur in the course of berry ripening, no detailed study on splicing modifications during this period has been published so far. We report here on the regulation of gene AS in developing berries of two grapevine (Vitis vinifera L.) varieties, Gewurztraminer (Gw) and Riesling (Ri), showing distinctive phenotypic characteristics. Using the software rMATS, the transcriptomes of berries at four developmental steps, from the green stage to mid-ripening, were analysed in pairwise comparisons between stages and varieties.

**Results:**

A total of 305 differential AS (DAS) events, affecting 258 genes, were identified. Interestingly, 22% of these AS events had not been reported before. Among the 80 genes that underwent the most significant variations during ripening, 22 showed a similar splicing profile in Gw and Ri, which suggests their involvement in berry development. Conversely, 23 genes were subjected to splicing regulation in only one variety. In addition, the ratios of alternative isoforms were different in Gw and Ri for 35 other genes, without any change during ripening. This last result indicates substantial AS differences between the two varieties. Remarkably, 8 AS events were specific to one variety, due to the lack of a splice site in the other variety. Furthermore, the transcription rates of the genes affected by stage-dependent splicing regulation were mostly unchanged, identifying AS modulation as an independent way of shaping the transcriptome.

**Conclusions:**

The analysis of AS profiles in grapevine varieties with contrasting phenotypes revealed some similarity in the regulation of several genes with developmental functions, suggesting their involvement in berry ripening. Additionally, many splicing differences were discovered between the two varieties, that could be linked to phenotypic specificities and distinct adaptive capacities. Together, these findings open perspectives for a better understanding of berry development and for the selection of grapevine genotypes adapted to climate change.

**Supplementary Information:**

The online version contains supplementary material available at 10.1186/s12870-021-03266-1.

## Background

The grape berry is a non-climacteric fruit produced for direct consumption and, predominantly, for wine-making, what makes viticulture an important economic activity in various regions of the world. However, the good growth and development of the grape berry are negatively impacted by climate change. Indeed, bud break, flowering and fruit-set occur earlier each year, while ripening takes place at higher temperatures, severely altering the biochemical composition of the berry [1]. Therefore, the control of fruit ripening is highly desirable in this context. A better knowledge of the molecular factors determining this developmental process might be useful for adapting our viticulture practices and breeding strategies to changing climate conditions [2]. Three main phases can be distinguished during berry development, i) a rapid growing phase involving cell division and cell expansion associated with tartrate and malate production, ii) a fleeting lag phase, iii) a ripening phase where glucose and fructose accumulate and aroma and flavors are biosynthesized [3]. The véraison marks the beginning of ripening, and is defined as the time when the berry starts to soften, and to accumulate colour pigments for black and red varieties. Transcriptional and proteomic profiling of developing berries have highlighted extensive changes in gene expression at different developmental stages [4–6]. Moreover, the exploration of an isoSeq-reconstructed transcriptome of Cabernet-Sauvignon berries showed that a major expression shift occurred at the onset of ripening, and also suggested that 25% of the transcribed genes could generate more than one splicing isoform [7]. On the whole, previous transcriptomic analyses have shown that berry growth and maturation involve important changes in the transcription rate of genes related to primary and secondary metabolism (amino-acids, organic acids, sugars, flavonoids), molecular transport, cell wall modification, and also to the synthesis of phytohormones and signaling [4–7]. Unsurprisingly, the development of this non-climacteric fruit is dependent on auxins and abscisic acid (ABA), which can be partly imported from the seeds to play complementary roles in the control of ripening [8–10]. Thus, auxins are crucial for cell division and berry growth before véraison, while ABA is essential to seed and berry maturation. The hormone content of seeds greatly determines the progression of berry ripening [11]. Moreover, the decrease in the level of auxin concomitant with the increase in the level of ABA in the pericarp, at the end of seed growth, seem to determine the ripening initiation of the fruit [8].

While the regulation of gene transcription in developing berries has been widely investigated, little is still known about the involvement of alternative splicing (AS) in the modulation of gene expression during this process. Occurring mainly in eucaryotes, AS is recognized as a widespread phenomenon contributing to protein diversity and regulation of gene expression. In plants, a majority of pre-messenger RNAs (pre-mRNAs) derived from multiexon genes are processed to produce splice variants with different fates and functions [12]. Loss- or gain-of-function can be engendered by the substraction/addition of a functional domain, changing the subcellular localization, the molecular binding characteristics or the activity of the mature transcripts or encoded proteins [13]. Frequently, AS generates transcripts with premature termination codons (PTCs) that can be targeted to the nonsense-mediated mRNA decay (NMD) pathway, although an incomplete mRNA can also be translated for regulating the function of the corresponding full-length protein [14–16]. RNA splicing occurs mainly co-transcriptionally, inside the nucleus, and is mediated by the spliceosome machinery involving many regulatory proteins [17]. The selection of constitutive and alternative splice sites is under the control of splicing factors, mainly serine-arginine rich (SR) proteins and heterogeneous ribonucleoproteins that bind cis-acting regulatory exonic/intronic splicing enhancers/silencers [18]. The regulation of AS modifies the ratio of different transcript isoforms that can be active in a specific tissue or at a particular developmental stage [13]. Light-induced regulation of AS plays a key role in plant morphogenesis, and the circadian clock, which orchestrates some major physiological processes, also regulates AS [19, 20]. In addition, differential splicing is highly responsive to stress factors, such as low or high temperature, high salt concentrations and water or nutrient deficiency, probably allowing for rapid adaptation to changing environmental conditions [21]. In grapevine, like in other plant species, these different stress factors have previously been shown to regulate gene AS [22–25]. SR genes, which play a major role in gene splicing, are themselves subjected to AS modulation, especially under stress conditions, and may regulate the splicing of multiple downstream target pre-mRNAs, at the same time, to modify the transcriptome [26, 27]. The extent of AS in grapevine has been first explored using cDNAs/ESTs collections available from public databases or merged RNAseq data to identify as many events as possible [22, 28]. The analysis of pooled RNAseq data from leaves, roots and berries exposed to various stress conditions, together with the comparison of two rootstock varieties, suggested that tissue, environmental conditions and even genotype may contribute to the diversity of AS profiles [22]. The study of the transcriptome of mature berries collected from ten grapevine varieties with various metabolic profiles further showed that more than half of the expressed multiexon genes produced several splice isoforms, the majority of which were conserved between varieties [29].

The following study was aimed at acquiring new knowledge about the factors underlying grape berry ripening, whose control is highly desirable in view of the adaptation of grapevine varieties to climate warming. Considering that differential splicing could play an important role in that developmental process, we analysed the regulation of AS in berries of two contrasting white *Vitis vinifera* L. varieties, Gewurztraminer (Gw) and Riesling (Ri). These two grapevine varieties present marked phenotypic differences, and their progenies show a strong variability that has previously been used for detecting QTLs linked to agronomic traits. Among these traits, the berries of Gw and Ri differ in color, sugar and aroma precursor contents [30–32], as well as in the concentration of several biochemical compounds (malic acid, tartaric acid, potassium ion) influencing their pH level [33, 34]. Moreover, the two varieties differ in phenology, as indicated by different dates of budbreak, flowering and véraison, the onset of ripening being generally delayed by about seven days in Ri compared to Gw [35]. We analyzed the regulation of AS in berries of Gw and Ri at four developmental stages, precisely defined: i) the early stage ‘hard green berry’, 6 weeks post-flowering (stage 1 = S1), ii) the stages 2 and 3 (S2 and S3) surrounding the véraison, berries being collected from clusters comprising about 50% of hard berries (S2) and 50% of soft berries (S3), iii) the mid-ripening stage (S4) defined based on the calculation of the heat sum of 230 °C.day after véraison, as previously described [35]. This approach allowed us to compare samples from the two varieties, at each stage, regardless of the speed of ripening progression for each variety. To closely track the variation of AS during berry development, our strategy consisted in the comparison of consecutive stages, representing crucial steps in the physiological development of the grape berry: first, a step of intense cellular division and expansion (from S1 to S2), then the shift to ripening (from S2 to S3), and finally a step of strong accumulation of sugars and secondary metabolites (from S3 to S4). The logical sequencing of the comparisons enabled us to consistently link the splicing variations detected to the biological processes occurring during the different phases. In addition, we statistically analysed the difference in gene AS between the two varieties at the different stages. The comparisons were performed using the software rMATS (replicate Multivariate-Analysis-of-Transcript-Splicing), primarily designed for AS analysis from replicate human samples [36], and that has recently been shown to be effective for accurate detection of differential AS (DAS) events in plant species [37].

## Results

### Total differential AS events detected in stage- and variety-comparisons

**Fig. 1:**
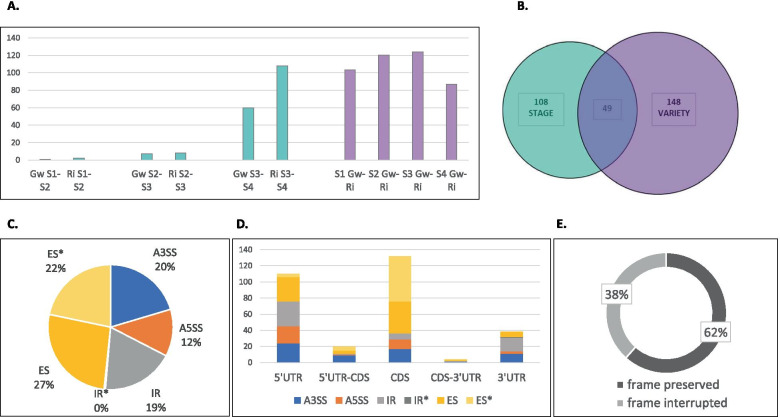
DAS events detected during berry development in Gw and Ri. **a** Number of differential events detected in each comparison between consecutive stages: S1 (green berry at 6 weeks post-flowering) vs S2 (hard berry at mid-véraison), S2 vs S3 (soft berry at mid-véraison) and S3 vs S4 (mid-ripening), as well as between the two varieties at each stage. **b** Relationship between the set of stage-regulated AS events and the set of AS events showing differential isoform ratios between Gw and Ri. **c** Distribution by AS type of the 305 unique DAS events identified in all comparisons: A3SS, A5SS, ES, IR, ES* and IR* (ES and IR not included in the VCost.v3 annotation). **d** Localization of the AS events of different types in the CDS or in the 3′- and 5′-UTRs. **e** Percentage of AS events localized in the CDS that preserve or not the reading frame

The comparisons between consecutive stages in Gw and Ri, and between the two varieties at each stage, were performed using replicates of RNAseq data obtained from berries harvested at four developmental stages, i.e. green berry, 6 weeks after flowering (S1), hard berry and soft berry at the mid-véraison stage (respectively, S2 and S3), and mid-ripening (S4). Four types of AS events were considered: usage of 3′- and 5′- alternative splice sites (A3SS and A5SS), exon skipping (ES) and intron retention (IR). For each AS event, the rMATS software distinguished two alternative isoforms and estimated the inclusion level (IL) as the proportion of the longest isoform among the total, which indicated the degree of AS. ILs were then automatically compared between two conditions to determine an IL difference (ILD) (significant at FDR ≤ 5%). In order to minimize the amount of the false positives, we selected the AS events supported by a minimum number of 15 reads of the rarest of the two isoforms, and presenting an IL between 10 and 90%, under at least one condition. On the whole, a maximum of 12% of all AS events were differential between two conditions, less than 1% in comparisons between S1 and S2 or between S2 and S3, and 5–12% in the other comparisons (Fig. 1a; Additional file 1). Most splicing variations were detected between the dates of mid-véraison and mid-ripening (from S3 to S4), in Gw and Ri, and between the varieties at each stage. A total of 305 unique differential events affected 258 different genes, aggregating 108 AS events which varied between consecutive stages and 148 between the two varieties, as well as 49 AS events varying at both stage and variety level (Fig. 1b). Among these, 67 AS events were newly identified with reference to the VCost.v3 genome annotation [38] (Canaguier et al. 2017). DAS events were divided into 49% of ES, 32% of A3SS and A5SS, as well as 19% of IR (Fig. 1c). They affected the coding sequence (CDS) or the 3′- or 5′-untranslated regions (UTRs) in proportions correlating well with the extent of these regions throughout the genome, as previously described [22] (Vitulo et al. 2014) (Fig. 1d). Moreover, the reading frame was preserved for 62% of the AS events affecting the CDS (Fig. 1e). The list, genomic coordinates and general description of these 305 DAS events are exhaustively reported in the Additional file 2. The results of gene ontology (GO) analysis performed on gene sets compiled either from stage or variety comparisons followed the same trends (Additional file 3). The most represented biological process (BP) categories were primarily ‘cellular/metabolic process’, and secondarily ‘biological regulation’, ‘localization’, ‘cellular component organization or biogenesis’ and ‘response to stimulus’. The classification by molecular function (MF) grouped them into the main categories ‘catalytic activity’ and ‘binding’, and secondarily ‘transporter activity’, while the classification by cellular component (CC) grouped them mainly into the categories ‘cell/cell part’, ‘organelle/organelle part’, ‘protein-containing complex’ and ‘membrane’. No category overrepresentation was observed among the genes showing AS variation between developmental stages, except for the BP category ‘macromolecule metabolic process’, although slightly (2.0 fold). By contrast, the list of genes showing splicing variation between the two varieties was enriched in several BP categories, the most noteworthy of them being related to ‘protein deacetylation’ (52.4 fold), ‘RNA polymerase III-mediated transcription’ (50.0 fold) and ‘splicing/alternative splicing’ (18.3 fold). Three other categories showed a weaker overrepresentation, i.e. ‘intracellular transport’ (3.6 fold), ‘gene expression regulation’, ‘metabolic process of nucleobases’, ‘RNA biosynthesis and metabolism’ (3.4 fold) and ‘primary metabolism regulation’ (3.0 fold). Enriched MF categories were related to ‘hydrolase activity’ (19.6 fold) and ‘RNA binding/binding’ (2.0–3.6 fold), while enriched CC categories were ‘histone deacetylase complex’ (34.4 fold), ‘nuclear speck/body/pore’ (10.6–20.4 fold), ‘nucleoplasm’ (7.8 fold) and other nuclear categories (2.4–4.4 fold), as well as ‘organelle’ and ‘membranes’ (2.0–4.0 fold). These results suggest that the two varieties substantially differed in gene expression regulation by AS, in the studied conditions. Additionally, the variation of AS was more pronounced, on average, when the two varieties were compared at any stage than when consecutive stages were compared for a single variety. Indeed, ILDs were higher in variety comparisons than in stage comparisons, and covered, respectively, a wider and a narrower range of values (−0.94 to +0.78 vs −0.50 to +0.41). Overall, 21.3% of the AS events distinguishing Gw and Ri showed ILD absolute values higher than 0.30, and 7.1% higher than 0.50, versus 14.0 and 0.6% of the AS events differentiating consecutive stages. However, 18% of all DAS events showed rather weak ILDs (|ILD| ≤ 0.15), that is, rather weak regulation rates. The following presentation focuses on 80 genes affected by at least one DAS event with an absolute value of ILD exceeding 0.15 in at least one condition, and for which consistent results were obtained in stage and variety comparisons.

### Genes showing similar splicing regulation in Gw and Ri during berry development

**Fig. 2:**
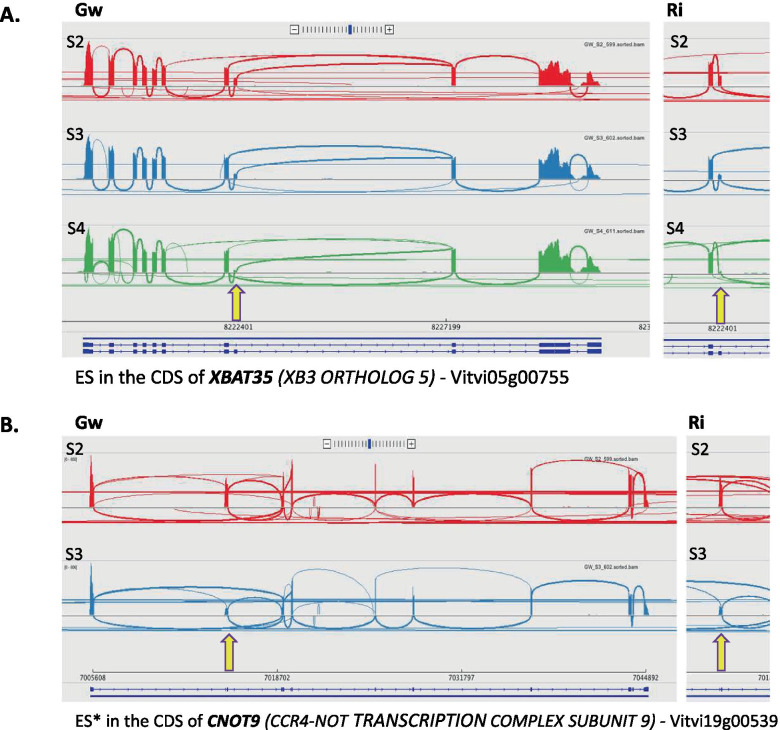
Similar regulation between Gw and Ri of an ES event in the CDS of (**a**) *XBAT35* and (**b**) *CNOT9*. The Sashimi plots showing RNAseq reads aligned to gene annotations at S2 (hard berry at mid-véraison), S3 (soft berry at mid-véraison) and S4 (mid-ripening) are respectively color-coded in red, blue and green. The arrows indicate the position of the skipped exons. The transcript variants included in the VCost.v3 annotation are presented as dark blue exon-plots: exons as solid lines and introns as dashed lines

**Fig. 3:**
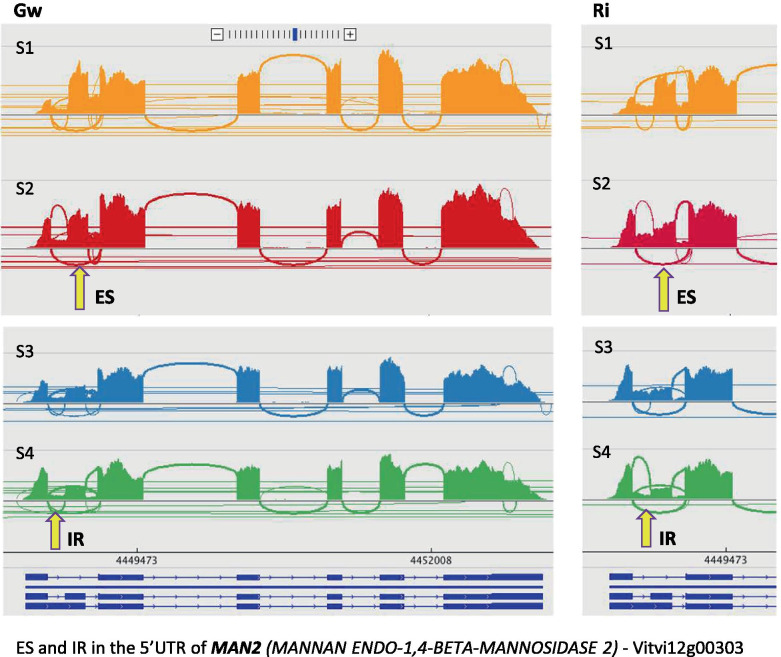
Similar regulation between Gw and Ri of two AS events in the 5’UTR of *MAN2*. Enhancement of an ES event between S1 (green berry at 6 weeks post-flowering) and S2 (hard berry at mid-véraison), followed by the down-regulation of an IR event between S3 (soft berry at mid-véraison) and S4 (mid-ripening). The Sashimi plots showing RNAseq reads aligned to gene annotations at S1, S2, S3 and S4 are respectively color-coded in orange, red, blue and green. The arrows respectively indicate the position of the skipped exon and of the retained intron. The transcript variants included in the VCost.v3 annotation are presented as dark blue exon-plots: exons as solid lines and introns as dashed lines

A number of genes underwent similar splicing regulation in Gw and Ri during the developmental phase investigated. Around the véraison time, however, only six genes were subjected to significant splicing variation (Table 1). Two ES events, respectively affecting the E3 ubiquitin-ligase gene *XBAT35* (Vitvi05g00755) and *CNOT9* (Vitvi19g00539) encoding a subunit of the deadenylation complex CCR4-NOT, were significantly amplified at the time of véraison (between S2 and S3), the ES in *XBAT35* being further intensified, significantly, between mid-véraison (S3) and mid-ripening (S4) (Fig. 2a, b). In fact, the proportion of the full-length transcripts progressively diminished until reaching a very low level at mid-ripening (S4) (IL_Gw_ = 0.09 and 0.05, and IL_Ri_ = 0.07 and 0.03, respectively for *XBAT35* and *CNOT9*). A third gene, *MAN2* (Vitvi12g00303) involved in the hydrolysis of mannan polysaccharides, was affected by two AS events located in the same region in the 5’UTR, which were regulated at two different times of the development process. First, an ES event was increased before véraison (between S1 and S2), then an IR event was down-regulated between mid-véraison (S3) and mid-ripening (S4), suggesting the production of three different isoforms in varying proportions at these different stages (Fig. 3). In addition, two AS events affecting two other genes, an E3 ubiquitin-protein ligase gene (Vitvi04g00220) of unknown function and *PRA1* (Vitvi08g01266) involved in intracellular vesicular trafficking, were similarly regulated in the two varieties at the véraison time (between S2 and S3), and even more strongly at mid-ripening (between S3 and S4) in Ri. The sixth gene, *RNP1* (Vitvi09g00196), a major actor in mRNA processing regulation, was subjected to the down-regulation of an IR event just before véraison in Ri and after véraison in Gw, highlighting a slight difference in splicing modulation between the two varieties. Seventeen additional AS events were similarly regulated between Gw and Ri after véraison (between S3 and S4) (Table 2). They affected genes with defined or hypothetical roles in development, transport, stress response or regulation of gene expression. In particular, these variations caused a shift in the balance of isoforms for *BLUS1* (Vitvi02g00218), *PP2C55* (Vitvi04g01249) and an FBD protein gene (Vitvi14g01295) (in Gw and Ri), as well as for *DAR1* (Vitvi04g02092) (only in Ri).

**Table 1:** Genes showing similar splicing regulation between Gw and Ri during the whole developmental period examined

			IL - Gw				ILD - Gw			IL - Ri				ILD - Ri		
Gene ID^a^	GENE NAME – biological process/function	AS^b^ (region)^c^	S1	S2	S3	S4	S1 vs S2 (FDR)	S2 vs S3 (FDR)	S3 vs S4 (FDR)	S1	S2	S3	S4	S1 vs S2 (FDR)	S2 vs S3 (FDR)	S3 vs S4 (FDR)
Vitvi04g00220	*E3 UBIQUITIN LIGASE*	**IR** (3’UTR)	_	0.20	0.35	_	**_**	**−0.15** (3.4 E-02)	**_**	_	0.15	0.30	0.69	**_**	**−0.15** (5.1 E-02)	**−0.40** (3.8 E-12)
Vitvi05g00755	***XBAT35*** – protein ubiquitination	**ES** (CDS)	_	0.38	0.22	0.09	**_**	**0.15** (6.9 E-02)	**0.14** (5.4 E-05)	_	0.41	0.22	0.07	**_**	**0.19** (2.7 E-04)	**0.15** (1.7 E-06)
Vitvi08g01266	***PRA1*** – intracellular transport	**A3SS** (3’UTR)	_	0.59	0.77	_	**_**	**−0.18** (3.3 E-03)	**_**	_	0.74	0.83	0.95	**_**	**−0.09** (1.6 E-02)	**−0.12** (1.3 E-03)
Vitvi09g00196	***RNP1*** – RNA processing/splicing	**IR** (5’UTR)	_	_	0.42	0.26	**_**	**_**	**0.16** (1.3 E-03)	_	0.54	0.35	_	**_**	**0.20** (2.0 E-03)	**_**
Vitvi12g00303	***MAN2*** *–* metabolic process	**ES** (5’UTR)	0.58	0.38	_	_	**0.21** (2.9 E-02)	**_**	**_**	0.53	0.33	_	_	**0.20** (2.2 E-02)	**_**	**_**
Vitvi12g00303	***MAN2*** *–* metabolic process	**IR** (5’UTR)	_	_	0.90	0.74			**0.16** (1.3 E-02)	_	_	0.87	0.65	**_**	**_**	**0.22** (5.6 E-03)
Vitvi19g00539	***CNOT9*** – transcription/translation	**ES** (CDS)	_	0.46	0.15	_	**_**	**0.32 (6.4 E-03)**	**_**	_	0.28	0.09	_	**_**	**0.19** (4.8 E-05)	**_**

The examined stages were S1 (green berry at 6 weeks post-flowering), S2 (hard berry at mid-véraison), S3 (soft berry at mid-véraison) and S4 (mid-ripening). The values of IL and ILD (significant at FDR ≤ 0.05) were means of three biological replicates

^a^according to the VCost.v3 genome annotation

^b^AS type, comprising A3SS, A5SS, ES and IR events

^c^region affected by the AS event (3′-UTR, 5′-UTR or CDS)

**Table 2:** Genes showing similar splicing regulation between Gw and Ri after the véraison time

			IL - Gw		ILD - Gw	IL - Ri		ILD - Ri
Gene ID^a^	GENE NAME – biological process/function	AS^b^ (region)^c^	S3	S4	S3 vs S4 (FDR)	S3	S4	S3 vs S4 (FDR)
Vitvi02g00218	***BLUS1*** - stomatal opening regulation	**ES** (CDS)	0.69	0.41	**0.29** (3.6 E-08)	0.72	0.28	**0.43** (0.0 E+00)
Vitvi04g00388	***MIEL1*** - control of cuticular wax synthesis	**IR** (5’UTR)	0.56	0.75	**−0.19** (4.5 E-06)	0.66	0.82	**−0.17** (1.7 E-02)
Vitvi04g01249	***PP2C55*** - stress signaling	**ES** (5’UTR)	0.16	0.53	**−0.37** (3.6 E-08)	0.21	0.67	**−0.46** (0.0 E+00)
Vitvi04g01506	***LARP1*** - mRNA stabilisation	**IR** (5’UTR)	0.82	0.66	**0.15** (1.1 E-02)	0.78	0.60	**0.18** (2.2 E-02)
Vitvi04g02092	***DAR1*** - cellular expansion regulation	**ES** (CDS)	0.91	0.70	**0.21** (3.8 E-09)	0.92	0.41	**0.51** (0.0 E+00)
Vitvi05g00358	***ATXN7*** - transcription regulation	**ES** (CDS)	0.94	0.76	**0.18** (7.3 E-03)	0.94	0.64	**0.30** (8.6 E-06)
Vitvi05g02099	***PUM6*** - mRNA stabilisation	**IR** (CDS)	0.85	0.62	**0.24** (6.4 E-04)	0.78	0.57	**0.21** (6.3 E-03)
Vitvi06g00267	*STORAGE PROTEIN-RELATED*	**ES** (5’UTR)	0.10	0.21	**−0.10** (8.9 E-02)	0.15	0.34	**−0.19** (2.1 E-05)
Vitvi06g00267	*STORAGE PROTEIN-RELATED*	**ES** (5’UTR)	0.34	0.59	**−0.25** (1.1 E-02)	0.47	0.74	**−0.27** (1.8 E-02)
Vitvi07g00031	***DRIP1*** - water stress regulation	**A5SS** (5’UTR)	0.84	0.65	**0.19** (1.4 E-02)	0.86	0.64	**0.22** (2.2 E-02)
Vitvi09g00606	***MBD11*** - organogenesis and development	**IR** (5’UTR)	0.91	0.75	**0.16** (1.5 E-05)	0.88	0.63	**0.25** (2.0 E-11)
Vitvi11g01416	***CID5*** *-* endoploidy regulation	**ES** (5’UTR)	0.57	0.40	**0.17** (6.6 E-04)	0.55	0.29	**0.26** (1.8 E-08)
Vitvi13g01256	***XPO7*** - nucleus to cytoplasm RNA transport	**IR** (5’UTR)	0.49	0.26	**0.24** (1.4 E-04)	0.27	0.12	**0.15** (7.8 E-02)
Vitvi14g01295	*FBD PROTEIN -* nuclear processes	**ES** (5’UTR)	0.39	0.59	**−0.20** (7.4 E-02)	0.25	0.68	**−0.43** (0.0 E+00)
Vitvi17g00561	***ABCA1*** - membrane lipid transport	**ES** (CDS)	0.82	0.50	**0.32** (1.7 E-05)	0.84	0.57	**0.27** (7.6 E-04)
Vitvi18g00597	***ITPK3*** - phytic acid synthesis	**ES** (CDS)	0.03	0.13	**−0.09** (4.5 E-02)	0.02	0.25	**−0.23** (0.0 E+00)
Vitvi18g00927	***LPCAT1*** - phospholipid biosynthesis	**IR** (CDS)	0.73	0.52	**0.22** (3.4 E-05)	0.69	0.49	**0.20** (1.9 E-02)

Splicing regulation occurred between S3 (soft berry at mid-véraison) and S4 (mid-ripening). The values of IL and ILD (significant at FDR ≤ 0.05) were means of three biological replicates

^a^according to the VCost.v3 genome annotation

^b^AS type, comprising A3SS, A5SS, ES and IR events

^c^region affected by the AS event (3′-UTR, 5′-UTR or CDS)

### Genotype-dependent variation of alternative splicing

**Fig. 4:**
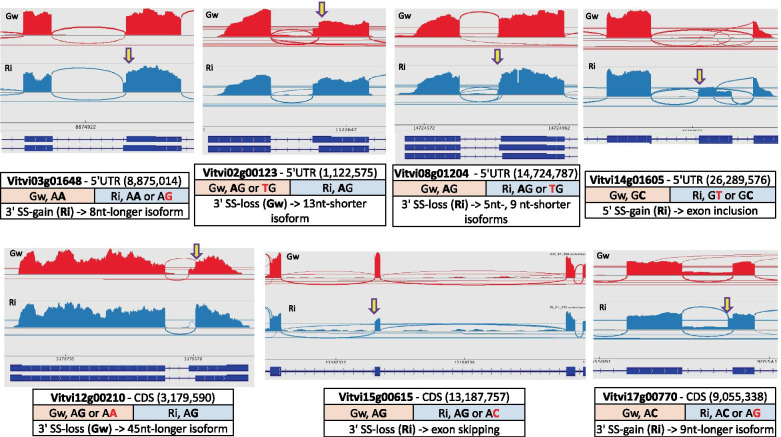
Variety-specific AS events associated with the gain or loss of a splice site. For each event, the arrow indicates the position of the SNP involved in the gain or loss of a splice site, by reference to the canonical GT (5′-donor) -and AG (3′-acceptor) sequences. The Sashimi plots showing RNAseq reads aligned to gene annotations are respectively color-coded in red for Gw and blue for Ri. The transcript variants included in the VCost.v3 genome annotation are presented as dark blue exon-plots: exons as solid lines and introns as dashed lines. The table under each plot summarizes, from top to bottom, first line: the gene ID, the region affected (3′-UTR, 5′-UTR orCDS), and the genomic location of the SNP, in brackets; second line: the nucelotide sequence at the position of the splice site in each variety (the specific nucleotide is in bold red font); third line: the splice site modification linked to the SNP and the specific alternative isoform (or specific event) produced

In contrast with the above-described cases, a number of splicing changes occurred in only one variety during the developmental period studied, suggesting a genotype-specific response to some internal or environmental stimuli. All these variations were detected between mid-véraison (S3) and mid-ripening (S4), except for an IR event affecting *CALR3* (Vitvi04g01198), a gene involved in the unfolded protein response pathway. This IR event was down-regulated in hard berries of Gw before mid-véraison (between S1 and S2), then recovered its initial level at the beginning of berry softening (S3 berries) (Table 3). Surprisingly, this AS event was not detected at all in the second variety, Ri. The other DAS events were of two types, depending on whether the ratios of isoforms were similar or not in the two varieties before mid-ripening (Table 4). Eleven events corresponded to the former case, for which differences in isoform ratios between Gw and Ri were only detected at mid-ripening (S4). The other case was represented by fifteen other AS events, for which splice isoform ratios distinguished Gw and Ri at S1, S2 and S3, five of them even being very specific to Ri (IL ≈ 0 or 1, in Gw) (Additional file 4). Finally, for a good number of additional AS events which were not significantly regulated between stages, we observed differential isoform ratios between Gw and Ri during the whole period of development studied (Table 5). Striking differences were represented by twenty-eight AS events uniquely or very predominantly dectected in one variety (IL ≈ 0 or 1, in the other variety), in addition to the six cases reported above. Seven other AS events were also remarkable due to the predominance of a different isoform in each variety (isoform shift). Among all AS events exclusively detected in one variety, eight were explained by a single nucleotide change leading to the suppression or the creation of a splice site, by reference to the canonical sequences GT (5′-donor) and AG (3′-acceptor) (Fig. 4). On the whole, the genes exhibiting variety-dependent splicing profiles were primarily related to the regulation of gene expression, to RNA processing and splicing, and secondarily to stress response and DNA damage repair. It is worth noting that, among important regulators of splicing, the SR genes *SCL30A* (Vitvi06g00073) and *RS40* (Vitvi15g00926) showed differential isoform ratios between Gw and Ri at each developmental stage.

**Table 3:** Successive up- and down-regulation of a Gw-specific IR event in the 3’UTR of *CALR3*

				IL - Gw			ILD - Gw	
Gene ID^a^	GENE NAME – biological process/function	AS^b^ (region)^c^	specificity	S1	S2	S3	S1 vs S2 (FDR)	S2 vs S3 (FDR)
Vitvi04g01198	***CALR3*** - unfolded protein response	**IR** (3’UTR)	ILs-Ri = 1	0.47	0.68	0.49	**−0.22** (0)	**0.19** (0)

The IR event was first increased between S1 (green berry at 6 weeks post-flowering) and S2 (hard berry at mid-véraison), then was diminished between S2 and S3 (soft berry at mid-véraison). The values of IL and ILD (significant at FDR ≤ 0.05) were means of three biological replicates

^a^according to the VCost.v3 genome annotation

^b^AS type, comprising A3SS, A5SS, ES and IR events

^c^region affected by the AS event (3′-UTR, 5′-UTR or CDS)

**Table 4:** AS events regulated between véraison and mid-ripening in only one variety

				IL - Gw		ILD - Gw	IL - Ri		ILD - Ri
Gene ID^a^	GENE NAME – biological process/function	AS^b^ (region)^c^	specificity	S3	S4	S3 vs S4 (FDR)	S3	S4	S3 vs S4 (FDR)
*Similar isoform ratios between Gw and Ri before mid-ripening*									
Vitvi01g00135	***VPS52*** - intracellular trafficking	**IR-1** (5’UTR)	_	0.27	0.12	**0.14** (3.2 E-03)	_	_	**_**
Vitvi01g00135	***VPS52*** - intracellular trafficking	**IR-2** (5’UTR)	_	_	_	**_**	0.54	0.76	**−0.22** (1.1 E-02)
Vitvi04g01976	***RSZ22*** - splicing regulation	**ES** (5’UTR)	_	_	_	**_**	0.32	0.15	**0.18** (2.8 E-10)
Vitvi05g00214	***BOR3*** - boron transport	**A3SS** (CDS)	_	_	_	**_**	0.77	0.52	**0.25** (5.9 E-06)
Vitvi05g00217	***RH40*** - NMD and ribosome biogenesis	**A3SS** (CDS)	_	_	_	**_**	0.42	0.22	**0.20** (1.8 E-02)
Vitvi09g01457	***ECT5*** - mRNA binding protein	**A3SS** (5’UTR)	_	_	_	**_**	0.54	0.77	**−0.24** (2.0 E-02)
Vitvi13g00175	***UXT3*** - xylose transport	**IR** (5’UTR)	_	_	_	**_**	0.24	0.44	**−0.20** (7.6 E-04)
Vitvi13g00613	*PHOSPHOGLUCOSAMINE MUTASE* - carbohydrate metabolism	**ES** (CDS)	_	_	_	**_**	0.55	0.25	**0.29** (2.5 E-07)
Vitvi18g01662	***STOP1*** - response to acidic stress	**ES** (5’UTR)	_	_	_	**_**	0.09	0.34	**−0.25** (1.8 E-07)
Vitvi18g00072	***PP2C27*** - abiotic stress modulation	**IR-1** (5’UTR)	_	0.78	0.52	**0.26** (1.5 E-05)	_	_	**_**
Vitvi18g00072	***PP2C27*** - abiotic stress modulation	**IR-2** (5’UTR)	_	0.88	0.73	**0.15** (7.9 E-03)	_	_	**_**
*Differential isoform ratios before mid-ripening*									
Vitvi01g00819	***NLRC3*** - intracellular signal transduction	**IR** (CDS)	ILs-Gw = 1	_	_	**_**	0.47	0.86	**−0.40** (6.7 E-04)
Vitvi01g02098	***SIN3A ISOFORM G*** - transcription regulator	**A3SS** (3’UTR)	_	_	_	**_**	0.96	0.79	**0.17** (5.9 E-05)
Vitvi02g00597	***CKB1*** *-* regulation of protein kinase activity	**A5SS** (CDS)	_	_	_	**_**	0.54	0.81	**−0.27** (0.0 E+00)
Vitvi06g01662	***S1FA1 -*** regulation of transcription	**A3SS** (5’UTR)	_	_	_	**_**	0.07	0.54	**−0.48** (6.7 E-03)
Vitvi13g01915	***AIMP1*** - tRNA aminoacylation	**A5SS** (CDS)	ILs-Gw = 1	_	_	**_**	0.65	0.91	**−0.26** (1.2 E-09)
Vitvi13g02138	***ARPP21*** - calmodulin-binding	**ES** (5’UTR)	_	0.55	0.74	**−0.20** (1.3 E-02)	_	_	**_**
Vitvi14g00256	*Unknown*	**ES** (CDS)	_	_	_	**_**	1.00	0.58	**0.41** (2.1 E-02)
Vitvi14g02541	*PROBABLE LIGASE*	**ES** (CDS)	ILs-Gw = 1	_	_	**_**	0.60	0.96	**−0.36** (6.9 E-12)
Vitvi15g00621	***p53*** - DNA damage repair	**IR** (3’UTR)	ILs-Gw ≤ 0.02	_	_	**_**	1.00	0.81	**0.19** (2.3 E-02)
Vitvi15g01145	***AHL9*** - DNA-binding transcription factor	**A5SS** (5’UTR)	_	0.80	0.95	**−0.16** (8.9 E-05)	_	_	**_**
Vitvi15g01145	***AHL9*** - DNA-binding transcription factor	**A3SS** (5’UTR)	_	0.80	0.95	**−0.15** (2.8 E-03)	_	_	**_**
Vitvi15g01145	***AHL9*** - DNA-binding transcription factor	**IR** (5’UTR)	_	0.88	0.97	**−0.09** (1.6 E-02)	_	_	**_**
Vitvi15g01173	***C1-THF synthase*** - folate synthesis	**ES** (CDS)	_	0.67	0.85	**−0.19** (1.0 E-03)	_	_	**_**
Vitvi17g00038	***CLPC1*** - protein translocation to chloroplast	**A3SS** (5’UTR)	_	_	_	**_**	1.00	0.78	**0.22** (1.1 E-02)
Vitvi17g00770	***WNK*** - response to stress	**A3SS** (CDS)	ILs-Gw ≤ 0.02	_	_	**_**	0.58	0.27	**0.31** (1.0 E-02)

The regulation occurred between véraison (S3: soft berry at mid-véraison) and mid-ripening (S4: mid-ripening). Similar or differential isoform ratios were observed between Gw and Ri before mid-ripening; ILDs between Gw and Ri at the different stages are given in the Additional file 4

^a^according to the VCost.v3 genome annotation

^b^AS type, comprising A3SS, A5SS, ES and IR events

^c^region affected by the AS event (3′-UTR, 5′-UTR or CDS)

**Table 5:** AS events showing differential isoform ratios between Gw and Ri with no significant regulation between stages

				ILD – Gw vs Ri			
Gene ID^a^	GENE NAME – biological process/function	AS^b^ (region)^c^	specificity	S1 (FDR)	S2 (FDR)	S3 (FDR)	S4 (FDR)
*Gw-specific or -preferential events*							
Vitvi02g00123	***DSPP*** *-* calcium ion binding	**A3SS** (5’UTR)	ILs-Ri = 1	**−0.43** (0)	**−0.40** (0)	**−0.39** (0)	**−0.35** (0)
Vitvi03g01316	***NUP160*** - nucleus-to-cytoplasm mRNAs transfer	**IR** (3’UTR)	ILs-Ri ≥ 0.97	**−0.47** (0)	**−0.42** (0)	**−0.43** (0)	weak expression
Vitvi04g01192	***KRI1*** - DNA damage-induced cell apoptosis	**ES** (3’UTR)	ILs-Ri ≤ 0.01	**0.36** (9.1 E-10)	**0.23** (1.4 E-05)	**0.16** (3.7 E-04)	**0.16** (2.6 E-05)
Vitvi04g01520	***GDAP2*** *-* growth and development	**IR** (3’UTR)	ILs-Ri = 1	**−0.62** (0)	**−0.52** (0)	**−0.56** (0)	**−0.52** (0)
Vitvi05g01796	***MEE*** - development regulation	**ES** (5’UTR)	ILs-Ri ≥ 0.97	**−0.53** (0)	**−0.50** (0)	**−0.49** (0)	**−0.33** (4.5 E-05)
Vitvi06g00189	***SAP5*** - environmental stress response	**A5SS** (5’UTR)	ILs-Ri ≤ 0.04	**0.40** (0)	**0.37** (0)	**0.32** (0)	**0.29** (0)
Vitvi12g00120	***ACD32.1*** - stress response	**A5SS** (CDS)	ILs-Ri ≤ 0.01	**0.30** (0)	**0.32** (0)	**0.27** (0)	**0.28** (0)
Vitvi12g00210	*unknown*	**A3SS** (CDS)	ILs-Ri = 0	weak expression	**0.37** (1.0 E-07)	**0.50** (2.5 E-14)	**0.30** (1.1 E-12)
Vitvi12g02216	***NRPB4*** *-* transcription initiation	**IR** (3’UTR)	ILs-Ri = 0	**0.59** (0)	**0.62** (0)	**0.57** (0)	**0.53** (0)
Vitvi14g01289	***SAMDC2*** *-* polyamines biosynthesis/homeostasis	**ES** (5’UTR)	ILs-Ri = 0	**0.58** (0)	**0.51** (0)	**0.55** (0)	**0.59** (0)
Vitvi14g02028	***GAUT 13*** *-* pectin/xylans biosynthesis	**ES** (CDS)	ILs-Ri = 1	**−0.23** (4.2 E-07)	**−0.17** (3.8 E-04)	**−0.24** (0)	**−0.15** (9.0 E-12)
Vitvi15g00615	***HIBCH1*** - amino acid catabolic process	**A5SS** (CDS)	ILs-Ri ≥ 0.98	**−0.34** (4.5 E-12)	**−0.36** (3.6 E-13)	**−0.34** (5.6 E-10)	weak expression
Vitvi15g01132	***FCF1*** *-* rRNA-processing & 40S assembly	**ES** (5’UTR)	ILs-Ri ≥ 0.99	**−0.15** (8.7 E-04)	**−0.14** (3.6 E-04)	**−0.18** (1.7 E-05)	**−0.16** (ns)
Vitvi15g01132	***FCF1*** - rRNA-processing & 40S assembly	**ES** (5’UTR)	ILs-Ri ≥ 0.98	**−0.15** (1.1 E-05)	**−0.17** (7.4 E-06)	**−0.20** (1.4 E-07)	**−0.29** (1.9 E-05)
*Ri-specific or -preferential events*							
Vitvi01g01659	***RANBP2*** - mRNA processing	**ES** (CDS)	ILs-Gw ≥ 0.98	weak expression	**0.15** (6.7 E-04)	**0.28** (0)	**0.14** (1.5 E-03)
Vitvi02g00836	***XLG3*** *-* receptor signaling pathway	**IR** (5’UTR)	ILs-Gw ≥ 0.99	**0.34** (0)	**0.30** (1.1 E-11)	**0.28** (8.7 E-10)	**0.36** (1.3 E-09)
Vitvi03g01648	*Unknown*	**A3SS** (5’UTR)	ILs-Gw ≤ 0.02	**−0.49** (0)	**−0.70** (0)	**−0.50** (0)	**−0.42** (0)
Vitvi04g01559	***c(3)G*** - DNA double-strand breaks repair	**A3SS** (5’UTR)	ILs-Gw ≥ 0.99	**0.39** (0)	**0.36** (0)	**0.41** (1.9 E-12)	**0.45** (0)
Vitvi04g02261	***MPPED2*** - unknown	**ES** (CDS)	ILs-Gw ≥ 0.99	**0.75** (0)	**0.85** (0)	**0.86** (0)	**0.64** (0)
Vitvi08g01204	***PBL11, NAK*** *-* growth and development	**A3SS-1** (5’UTR)	ILs-Gw = 1	**0.54** (6.7 E-14)	**0.46** (0)	**0.47** (ns)	weak expression
Vitvi08g01204	***PBL11, NAK*** *-* growth and development	**A3SS-2** (5’UTR)	ILs-Gw = 1	**0.42** (ns)	**0.28** (1.7 E-06)	**0.47** (ns)	weak expression
Vitvi11g00574	***FBL14*** *-* ubiquitin-dependent protein catabolism	**A3SS** (5’UTR)	ILs-Gw = 1	**0.22** (0)	**0.25** (0)	**0.20** (0)	**0.26** (0)
Vitvi11g00614	***EF1B*** *-* translation elongation	**A3SS** (5’UTR)	ILs-Gw = 1	**0.34** (9.7 E-12)	**0.36** (3.9 E-11)	**0.49** (1.3 E-12)	weak expression
Vitvi11g00614	***EF1B*** - translation elongation	**ES** (5’UTR)	ILs-Gw ≥ 0.97	**0.54** (0)	**0.36** (2.0 E-03)	**0.57** (0)	**0.77** (0)
Vitvi11g01128	***GCNT*** *–* transfer of glycosyl groups	**A3SS** (5’UTR)	ILs-Gw ≤ 0.02	**−0.27** (4.1 E-14)	**−0.34** (6.9 E-12)	**−0.31** (2.2 E-10)	weak expression
Vitvi12g02011	***RPPL1*** *-* disease resistance	**IR** (3’UTR)	ILs-Gw ≥ 0.93	**0.34** (3.0 E-04)	**0.32** (2.2 E-05)	**0.41** (3.1 E-14)	**0.42** (4.3 E-12)
Vitvi14g01605	***G6PD*** *-* glucose metabolic process	**ES** (5’UTR)	ILs-Gw = 0	**−0.16** (0)	**−0.18** (1.6 E-13)	**−0.18** (0)	**−0.18** (0)
Vitvi15g00615	***HIBCH1*** *-* branched-chain amino acid catabolism	**ES** (CDS)	ILs-Gw = 1	**0.29** (3.7 E-10)	**0.31** (5.7 E-10)	**0.27** (1.4 E-06)	weak expression
*Other differential events*							
Vitvi01g00383	***MAF1*** - repression of transcription	**A3SS** (5’UTR)	Isoform shift	**0.44** (0)	**0.45** (0)	**0.43** (1.2 E-13)	**0.45** (3.3 E-10)
Vitvi03g00257	***ACY1*** - hydrolysis of N-acylated amino acids	**A3SS** (CDS)	_	**0.16** (0)	**0.16** (0)	**0.16** (0)	**0.14** (9.5 E-06)
Vitvi06g00073	***SCL30A*** - regulation of mRNA splicing	**ES** (CDS)	Isoform shift	weak expression	**0.27** (ns)	**0.32** (6.2 E-03)	weak expression
Vitvi06g00216	***DTX42*** - aluminium tolerance	**A5SS** (5’UTR)	Isoform shift	**0.56** (0)	**0.39** (9.7 E-10)	**0.42** (1.9 E-04)	**0.30** (1.3 E-03)
Vitvi07g01499	***GCL1*** - carbohydrate metabolic process	**ES** (CDS)	_	**−0.30** (1.4 E-07)	**−0.28** (2.0 E-04)	**−0.24** (1.9 E-09)	**−0.32** (0)
Vitvi09g01389	***PUM5*** - regulation of translation	**A3SS** (3’UTR)	Isoform shift	weak expression	**0.31** (2.3 E-05)	**0.24** (1.2 E-03)	**0.29** (4.5 E-05)
Vitvi10g00052	***OSCA1*** - response to osmotic stress	**A5SS** (5’UTR)	Isoform shift	**−0.30** (1.1 E-08)	**−0.25** (8.5 E-04)	**−0.27** (1.0 E-04)	**−0.35** (0)
Vitvi10g00094	***SS3*** - starch biosynthesis	**A5SS** (5’UTR)	_	**−0.22** (1.5 E-03)	**−0.27** (5.7 E-04)	**−0.28** (1.4 E-05)	**−0.21** (ns)
Vitvi10g00577	*HYDROLASE-LIKE PROTEIN FAMILY*	**A5SS** (3’UTR)	Isoform shift	**0.29** (1.0 E-02)	**0.21** (ns)	**0.32** (7.5 E-04)	**0.32** (6.9 E-03)
Vitvi11g00473	*RIBOSOMAL PROTEIN* - translation	**ES** (5’UTR)	_	**0.13** (1.9 E-03)	**0.20** (2.5 E-13)	**0.16** (5.9 E-09)	**0.14** (4.1 E-05)
Vitvi15g00926	***RS40*** – RNA/mRNA splicing	**ES** (5’UTR)	Isoform shift	**0.34** (3.0 E-03)	**0.23** (ns)	**0.34** (3.6 E-10)	**0.23** (1.7 E-06)

AS events were split in variety-specific or -preferential events (Gw or Ri) and other differential events. The examined stages were S1 (green berry at 6 weeks post-flowering), S2 (hard berry at mid-véraison), S3 (soft berry at mid-véraison) and S4 (mid-ripening). The value of ILD (significant at FDR ≤ 0.05) was a mean of three biological replicates

^a^according to the VCost.v3 genome annotation

^b^AS type, comprising A3SS, A5SS, ES and IR events

^c^region affected by the AS event (3′-UTR, 5′-UTR or CDS)

### Analysis of the expression of DAS genes at the transcriptional level

Subsequently, we examined the transcription rate of the genes that underwent AS regulation in the course of berry development in Gw and Ri. Within the group of 140 genes affected by 157 AS events variously regulated between consecutive stages, only 25 were also regulated at the transcriptional level (log_2_ fold change (FC) ≥ 1.0), with a medium intensity of ca. 2–3 fold and a limited temporal overlap of the two types of regulation (Additional file 5). Among the genes above noticed for undergoing remarkable splicing regulation (Tables 1, 2, 3 and 4), *MIEL1* (Vitvi04g00388) was subjected to similar transcriptional up-regulation in the two varieties (3-fold and 2.6-fold, respectively) between mid-véraison (S3) and mid-ripening (S4), simultaneously to the up-regulation of an IR in the 5’UTR of the gene. In addition, the transcription rates of *BOR3* (Vitvi05g00214) and of another gene of unknown function (Vitvi14g00256) were respectively decreased (2.9-fold) and increased (2.3-fold) in Ri, between S3 and S4, at the same time as the regulation of an AS event. By searching for regulation events of lower-intensity (0.6 ≤ log_2_ FC ≤ 1.0), we found that the transcription of *MAN2* (Vitvi12g00303) was slightly down-regulated between the two green-berry stages S1 and S2, in Gw and Ri (respectively, 1.5–1.6 fold), when an ES in the 5′-UTR of the gene was simultaneously enhanced. Likewise, the transcription of *ATXN7* (Vitvi05g00358) was slightly up-regulated (1.6 fold) in Gw and Ri, between mid-véraison and mid-ripening, in parallel with the regulation of an ES event in the CDS. In addition, *VPS52* (Vitvi01g00135), *LARP1* (Vitvi04g01506), *RSZ22* (Vitvi04g01976), *RH40* (Vitvi05g00217) and *PUM6* (Vitvi05g02099) were weakly regulated at the transcriptional level (1.5–1.8 fold) in Ri, between S3 and S4, just when a splicing event was also regulated. In summary, very few of the genes affected by splicing variation during the studied period were concomitantly regulated at the transcriptional level, highlighting a lack of correlation between these two biological processes.

## Discussion

### Efficient and accurate detection of splicing regulation during berry ripening

Our study was aimed at identifying AS variation during berry ripening, in two white grapevine varieties, Gw and Ri, that typically show differences related to phenology, as well as berry morphology and metabolism [30–35]. Four developmental stages surrounding the crucial moment of véraison, at the start of fruit ripening, were investigated. The DAS events detected in this study composed only a fraction (≤ 12%) of the total of all AS events retained in each comparison, reflecting that AS mostly acted without being regulated during the period examined, and essentially produced transcript isoforms in similar proportions in the two varieties. However, a total of 305 unique AS events presented differential isoform ratios in the ten pairwise comparisons performed, one half in stage comparisons and the other half in variety comparisons. Remarkably, almost one-quarter of these AS events had not been previously mentioned in the reference VCost.v3 genome annotation. Despite the great interest in deciphering fruit development mechanisms in crop species, few similar studies have been reported until now. Moreover, some discrepancies appear, especially in the total number of differential AS events detected. These inconsistencies seem to be mostly attributable to the various levels of performance of the methods used for AS event detection and validation. For instance, splicing analysis during the course of blueberry fruit development highlighted more than 700 genes affected by AS regulation when using the Cuffdiff program, while the use of the ArabiTag algorithm enabled the identification of only ca. 90 regulated genes [39]. It is now accepted that increased sequencing depth, together with the consideration of multiple reads from replicate samples covering a splice junction, allow for improving the accuracy of AS event detection. We used the rMATS software, shown effective in plants for accurate AS analysis from replicate data [37], to analyze and statistically validate the differential events occurring between two conditions, either consecutive stages, or the two varieties at each stage. Thereafter, we applied stringent threshold values for the number of reads covering the splice junction and for the ratio of splice isoforms (ILs), in order to select only the most reliable results. Indeed, the number of AS events can be overestimated when taking into account the many low abundant and incompletely spliced (IR) transcripts constituting a noise in the splicing process [40]. Remarkably, our analysis highlighted many differential exon skipping/inclusion events (≈ 50%), that have often been described as the least frequent AS events occurring in plants, although this assertion was mainly based on studies in Arabidopsis [12, 41]. The investigation of AS in other plant species suggest that this type of event occurs much more frequently than generally reported. For instance, ES events were found to be only 1.5 fold less frequent than IR events in maize leaves [42] and slightly more frequent in the kiwi fruit [43]. These findings argue in favor of a sofar underestimated importance of ES events in plants. It is worth mentioning that almost half of the differential ES events detected by rMATS were newly identified AS events, suggesting that this software was particularly well-suited to this task.

### Splicing regulation of a set of genes putatively involved in fruit development

Among all AS events regulated between successive developmental stages, many varied between mid-véraison (S3) and mid-ripening (S4), and only a few ones between the two first stages S1 and S2, and between the stages hard-berry (S2) and soft-berry (S3) at the time of mid-véraison. This result was rather unexpected in view of the profound metabolic modifications and extensive transcriptional reshaping usually observed at the onset of véraison [4–7]. While no particular GO term enrichment was found in the set of genes affected by AS events regulated between developmental stages, a special look to the most significant events showed that they mainly impacted some genes putatively involved in gene expression regulation, stress response and development (cf. Tables 1, 2, 3 and 4). Fruit formation is associated with numerous changes at the morphological and physiological levels and involves an intense metabolic activity. Seeds develop at the same time as the pericarp, and reach their maximum peak volume at véraison, progressively accumulating carbohydrate and lipid reserves before entering dessication during the second phase of berry ripening [11]. The stage-regulated AS events that were shared by the two varieties held our attention due to a possible role in berry development. To our knowledge, only the ES occurring in the pre-mRNA of *XBAT35* (Vitvi05g00755) has already been assigned a specific function, at the moment. The skipping of the exon-8 has been characterized in Arabidopsis and shown to produce a nuclear localization signal (NLS)-lacking variant, XBAT35.2, consistently localized in the cytoplasm, in contrast with the nuclear localization of the full-length transcript XBAT35.1 [44]. The E3 ligase isoform XBAT35.2, functioning in the ubiquitin-proteasome system by targeting specific substrates, has been shown to regulate the morphogenesis of Arabidopsis plantlets [44] and is thought to play dual roles in biotic and abiotic stress responses [45, 46]. Interestingly, when Arabidopsis young plants were treated with the phytohormone ABA, the transcription and translation of XBAT35.2 were induced, negatively controlling the tolerance to abiotic stress [46]. Given the fact that ripening of the grape berry, a non-climacteric fruit, is highly dependent on ABA, the increasing predominance of XBAT35.2 from pré-véraison to mid-ripening could have a particular significance in this context. Several other genes affected by similar splicing regulation in the two varieties, during berry ripening, deserve attention. For instance, *MAN2* (Vitvi12g00303), involved in the hydrolysis of mannan polysaccharides which are structural components of the plant cell wall, was subjected to AS regulation before the start of berry softening and maturation, and secondarily, at the mid-ripening stage which is characterized by changes in osmotic pressure accompagnying cell wall disassembling. In parallel, we measured a slight down-regulation of its transcription between S1 and S2 in Gw and Ri. This gene has previously been shown to be transcriptionally regulated during grape berry development, and its expression could be linked to the maintenance of cell wall relaxation and pericarp elasticity in the course of ripening [47, 48]. It is tempting to speculate that the differential splicing occurring in the 5’UTR of *MAN2* could impact its expression, since this region of the mRNA is known to drive the efficiency of translation which may vary according to its sequence and conformation [49]. In addition, *PRA1* (Vitvi08g01266), belonging to a gene family encoding Rab receptors preferentially expressed in developing tisssues [50], was subjected to AS regulation from the moment of véraison. This gene most probably regulates the trafficking of intracellular vesicles, a process tied to cell wall remodelling and textural changes during fruit maturation [51]. Likewise, the splicing regulation of *MIEL1* (Vitvi04g00388) coding an E3-ubiquitin ligase involved in cuticular wax biosynthesis [52] could be significant in this context, particularly given that we observed its transcriptional up-regulation at the same time. Waxy polymers confer hydrophobicity to the outer layer of the fruit skin, and their amount and composition have recently been shown to be modulated during berry development [53]. Their synthesis requires fatty acids whose metabolism has been shown to be transcriptionally regulated during the biogenesis of the grape berry exocarp [54]. We also detected the AS regulation of *LPCAT1* (Vitvi18g00927) and *ABCA1* (Vitvi17g00561), respectively encoding a lysophosphatidylcholine acyltransferase catalysing the biosynthesis of lipids and a membrane lipid transporter, as well as of *ITPK3* (Vitvi18g00597) belonging to a small gene family involved in the synthesis of phytic acid, which is an essential phosphore-storage component of plant seeds. In addition, the AS regulation of *CID5* (Vitvi11g01416) coding for a polyadenylate-binding-protein-interacting protein probably involved in endoploidy regulation could be linked to berry growth, as suggested by endoreduplication studies in tomato [55]. Moreover, *DAR1* (Vitvi04g02092) a regulator of cellular endoreplication supposed to control seed size and number [56] underwent splicing regulation in the two varieties between véraison and mid-ripening, although more strongly in Ri.

### Stress-related genes showing splicing variation between véraison and mid-ripening

Splicing regulation was also found to affect some stress-related genes, at the transition point between véraison and mid-ripening, suggesting that grape berries activated their stress response pathways during this period. In particular, *BLUS1* (Vitvi02g00218) encoding a ser/thr protein present in guard cells and phosphorylated by phototropins for blue-light dependent stomatal opening [57], as well as a putative stress-related PP2C55 encoding gene (Vitvi04g01249), were affected by ES events strongly regulated in the two varieties. Additionnally, an AS event was regulated in the 5’UTR of *DRIP1* (Vitvi07g00031) encoding a RING E3 ligase known to interact with DREB2A for controlling the expression of drought-responsive genes [58]. Several other genes probably involved in the response to abiotic stresses were regulated between véraison and mid-ripening, i) only in Gw, such as *PP2C27* (Vitvi18g00072), a drought responsive gene, or ii) specifically in Ri, such as *STOP1* (Vitvi18g01662) involved in the tolerance to acidic stress, *BOR3* (Vitvi05g00214) potentially essential for cell wall maintenance [59] as well as *CKB1* (Vitvi02g00597) involved in the response to UV-B stress [60]. The genes belonging to the stress response pathways seem to be particularly prone to AS regulation, which is thought to be a way to promptly change the transcriptome in response to challenging environmental conditions [61]. Although grapevine plants generally tolerate moderate drought conditions, extreme light and temperature combined with water scarcity may negatively impact the development of varieties adapted to temperate climates [62]. The warm and dry conditions encountered in summer, in our geographical region, could explain a marked stress-response of the varieties between véraison and mid-ripening, notably of Ri, which is particularly sensitive to drought and UV-irradiation.

### Divergence in the splicing of 168 genes between Gw and Ri

The most unexpected finding of this study was the differential AS profiles exhibited by the two grapevine varieties. When gene AS was compared between Gw and Ri at each developmental stage, a total of 197 differential events affecting 168 different genes were detected. The most distinctive AS events were events occurring specifically or very preferentially in a single variety (cf. Tables 4 and 5, Supplemental file 4). GO enrichment analysis suggested that the two varieties particularly differed in the splicing of genes related to transcription and translation regulation as well as to RNA splicing, itself. Interestingly, the SR protein genes *SCL30a* (Vitvi06g00073) and *RS40* (Vitvi15g00926) showed differential isoform ratios between Gw and Ri at all developmental stages. Belonging to the splicing machinery, SR proteins regulate constitutive and alternative RNA splicing, and are known to be themselves subjected to AS regulation [41]. These versatile proteins are present in the cell, at various phosphorylation degrees, to induce the splicing of multiple downstream genes in response to developmental requirements or to changing environmental conditions [63]. Particularly, splicing modulation of *SR* genes seems to be largely induced by stress factors. *SCL30a* (Vitvi06g00073) and *RS40* (Vitvi15g00926), that belong to the plant specific SC35/SCL sub-family, are responsive to ABA-mediated stress [64]. In addition, *RS40* is involved in the biogenesis of micro-RNAs (mi-RNAs) which play a substantial role in gene expression regulation [65]. The genotype-dependent splicing variation of *SR* genes has already been reported. In the genus *Vitis*, Vitulo et al. [22] observed that the proportions of splice variants of several *SR* genes differed strikingly between two rootstock genotypes. Furthermore, many *SR* transcript isoforms were found to be ‘species-specific’ in a study comparing maize and sorgho [66]. Several previous reports have also mentioned the overrepresentation of genes involved in RNA splicing among genes showing splicing variation depending on the developmental stage, the environment, or the genotype, in various plant species [27, 39, 67, 68]. We found that a number of genes related to the response to nutritional or environmental stresses were among those with the most marked splicing differences between Gw and Ri. For instance, the homolog of the human p53 gene (Vitvi15g00621), a negative regulator of cell proliferation in response to stress and DNA-damage, showed particularly contrasted splice isoforms in the two varieties, as well as, at a lesser degree, *KRI1* (Vitvi04g01192) and *c(3) G* (Vitvi04g01559) also active in the DNA-damage response. This could reflect differences between the two varieties in the way, or the capacity, to face stress conditions. The most extreme divergence between Gw and Ri consisted of AS events detected solely in one variety. For eight out of sixteen such events, the specific isoform was associated with an SNP determining the presence/absence of a splice site. Splice-site gain and loss have already been observed in comparisons of various plant species [69]. Furthermore, domesticated and wild sunflowers were found to differ in more than 100 species-specific AS events, suggesting a link between the differentiation of AS and the mechanism of speciation [70]. In the present study, the detection of splicing variation at the intraspecific level raises the question of the diversity and evolution of gene AS in grapevine, a highly heterozygous species mainly propagated by vegetative methods.

### Significance of the AS events highlighted by comparisons of developmental stages and varieties

Except for the exon8-skipping in the pre-mRNA of the E3 ubiquitin ligase *XBAT35* (Vitvi05g00755), none of the splicing events reported here has been linked to a precise function in the plant cell. Actually, relatively little is known about the real importance and biological meaning of the multiple isoforms detected through splicing analysis in plants. When an AS event includes/excludes a protein domain in the CDS, an alternative functional protein isoform may be produced, whereas the introduction of a PTC often leads to an unproductive isoform, either interfering with the functional transcript for its localization or stability, or being decayed by a cell surveillance mechanism [12, 41]. Untranslated regions of pre-mRNAS, as well, undergo AS events, with great potential impact on gene expression. Indeed, some modifications of the 5’UTR sequence have been shown to efficiently modulate mRNA translation [71]. Riboswitches involved in local conformation changes after binding with regulatory molecules, as well as short upstream Open Reading Frames, may attenuate or speed up translation and can be modified by AS [49]. In addition, differential splicing can modify miRNA-binding sites, especially frequent in 3’UTRs, to change the transcriptome in response to internal or external stimuli [42, 72]. Among all genes found to be regulated at the splicing level in this study, many were involved in transcription, RNA processing and splicing, as well as in ribosome biogenesis and in translation, in accordance with previous observations on the main functional categories to which genes that are regulated by AS belong [73]. Strikingly, we observed a poor correlation between the transcription rate and the AS regulation profile of these genes, suggesting that, most of the time, only isoform ratios were modified. This is consistent with the fact that the subset of DAS genes generally weakly overlap with the subset of genes regulated at the transcriptional level, the two subsets also exhibiting distinctive features in DNA methylation and distribution of regulatory sequences [73]. A number of AS events described in this study were similarly regulated in the course of fruit ripening in Gw and Ri, which leads to the hypothesis that they might have a genuine role in this process. In particular, the conservation of the exon8-skipping event affecting *XBAT35* in different plant species, and its similar up-regulation in Gw and Ri from the green-berry stage to mid-ripening, are remarkable. Conversely, many AS events were found to distinguish the two varieties and might contribute to phenotypic variation, as Gw and Ri present specificities at the phenological, morphological and metabolical level. Nevertheless, all the AS events reported above deserve further examination to identify those who have real operating functions during berry ripening or could determine variety-specific characteristics.

## Conclusions

We report here, for the first time, a precise analysis of AS regulation in the course of grape berry ripening, from the green stage, six weeks post-flowering, to mid-ripening. On the whole, 305 unique DAS events, affecting 258 different genes, were detected in stage- and variety- comparisons. More than 20% of these AS events had not been previously reported, stressing the relevance and accuracy of our analysis. Half of all pinpointed AS events were regulated between consecutive developmental stages. Some of them were similarly regulated in the two varieties, strongly suggesting that they could be essential factors in the process of berry ripening. Alternatively, several AS events that varied between stages in only one variety, are potentially linked to variety-specific responses to developmental or environmental constraints during this period. We found that the regulation of AS was weakly correlated with that of the transcription rate, suggesting that, most often, only the ratios of transcript isoforms were modified. This observation strengthens the emerging view that AS is an independent way of transcriptome reprofiling in response to specific internal or external necessities. The other half of all DAS events detected in this study revealed differences in isoform ratios between Gw and Ri without regulation in the course of ripening. On the whole, a large and rather unexpected divergence in splicing patterns was discovered between the two varieties, especially highlighted by a set of 34 variety-specific or -preferential AS events, some of which were explained by the gain or loss of a splicing site, donor (5′) or -acceptor (3′). These specificities could underly distinctive phenotypic and adaptive characteristics for the two varieties. The role of the many genes found to undergo splicing variation in this study have poorly been described in plants, and even less the functions of the different splice isoforms. Their further characterization will certainly be useful for improving our understanding of the process of berry ripening. Moreover, complementary investigations on the AS events distinguishing Gw and Ri could help explaining distinctive phenotypic characteristics, and also maybe disparities in the ability for the two varieties to face hard environmental conditions during warm and dry periods. Further studies should therefore open perspectives for the selection of genotypes adapted to climate change.

## Methods

### Plant materials

This study complies with relevant institutional, national, and international guidelines. Plants of the *Vitis vinifera* L. varieties and of the *Vitis* rootstock were obtained from our own repository (INRAE-Grand Est, Colmar). The scion varieties Gw (clone 643) and Ri (clone 49) were grafted onto the rootstock 161–49 Couderc (clone 198), then grown in an experimental vineyard settled in Bergheim-France, as already described [35]. Temperatures were recorded at a weather station located nearby the experimental vineyard. For DNA analysis, young open leaves were collected from five plants of each cultivar in may 2013, weighed and immediately frozen in liquid nitrogen before extraction. For RNA analysis, whole berries were collected from the two cultivars in 2016, at four developmental stages. Berries at the firt stage (S1) were hard green berries collected at 6 weeks post-flowering. Stage-2 (S2) and stage-3 (S3) berries were respectively hard and soft berries, harvested at the time of mid-véraison, corresponding to the onset of ripening and indicated by the softening of the pericarp. The véraison is a fleeting step and not all berries reach this stage exactly at the same time in the same grape cluster; the complete shift to ripening can take a few days (2–4). Berries at S2 and S3 were collected from clusters comprising about 50% of hard berries (S2) and 50% of soft berries (S3). The stage of each berry was determined by palpation. The stage 4 (S4) is the mid-ripening stage. The determination of the date for collecting S4 berries was based on the daily recording of the ambient temperature after véraison, the heat sum of 230 °C.day being established as the threshold to meet for the mid-ripening stage, as previously described [35]. For each sampling, berries were collected from grape clusters at the right developmental stage on five plants of each experimental block, three blocks forming three biological replicates. Intact fresh berries were immediately frozen in liquid nitrogen and stored at −80 °C before nucleic acid extraction.

### DNA extraction

DNA was extracted from 3.5 g of fresh intact leaves, frozen, then crushed in liquid nitrogen. Cell nuclei were first purified. The powdered tissue was suspended in 45 mL of Sucrose Extraction Buffer (SEB) containing 0.01 M Tris, 0.1 M KCl, 0.01 M EDTA (0.5 M, pH 8), 0.55 M sucrose, 5 mM spermidine, 0.13% carbamic acid, 0.25% PVP 40, and 0.2% β-mercaptoethanol, before incubation on ice for 12 min. The supernatant was filtered through Miracloth (475,855, Merck Millipore®, France) then through a cell strainer (40 μm, Falcon®, Germany) and incubated on ice for 12 min before adding 0.15 vol. of SEB with 10% triton (X 100). Incubation on ice was continued for 12 min before centrifugation (600 g, 10 min, 4 °C). The pellet was resuspended in 60 mL of SEB for an additional filtration on a cell strainer (40 μm, Falcon®, Germany) before incubation on ice and centrifugation in the same conditions. The pellet was resuspended in 2 mL of SEB. Nuclei were lysed by adding 3 vol. of TBL buffer containing 0.4 M EDTA (0.5 M, pH 8), 2% N lauryl sarkosyl and 1 mg.mL^−1^ Proteinase K, before incubation at 55 °C for 3 h with gentle shaking. DNA extraction and purification were performed using the DNeasy™ Plant Maxi kit (Qiagen, France) following the manufacturer’s recommendations. DNA extracts were stored at −20 °C.

### RNA extraction

Total RNA was extracted from 1 g of frozen berries crushed in liquid nitrogen. The powdered tissue was suspended in 15 mL of pre-warmed (65 °C) buffer containing 2% CTAB, 2% PVP, 0.1 M Tris (1 M, pH 8), 2 M NaCl, 25 mM EDTA (0.1 M, pH 7.5), 0.5 g.L^−1^ spermidine and 2% β-mercaptoethanol, before incubation at 65 °C for 10 min. After centrifugation (5000 g, 15 min, 4 °C), the supernatant was treated by adding 1 vol. of a mix of chloroform:isoamyl alcohol (24:1), before centrifugation (5000 g, 15 min, 4 °C) to eliminate the proteins in the pellet. This last step was reiterated. Nucleic acid precipitation was performed by adding 0.1 vol. of sodium acetate (3 M, pH 5.2) and 0.6 vol. of isopropanol to the aqueous phase, before incubation at −80 °C for 30 min. After centrifugation (9500 g, 30 min, 4 °C), the pellet was resuspended in 1 mL TE buffer (1 X, pH 7.5). RNA precipitation was performed by adding 0.3 vol. of sterile LiCl (8 M) before incubation at 4 °C for the night. After centrifugation (16,000 g, 30 min, 4 °C), the pellet was whashed with 500 μL of 70% ethanol, centrifuged in the same conditions, then dried at room temperature. The pellet was resuspended in RNase-free water before DNA hydrolysis using the DNase TURBO™ (ThermoFischer scientific, France) following the manufacturer’s recommendations. The extract (50 μL) was purified by dilution in 4 vol. of RNase free water and 5 vol of phenol:chloroform:isoamyl alcohol (25:24:1). After centrifugation (16,000 g, 5 min, 20 °C), the supernatant was treated with 0.5 vol. of chloroform and centrifuged in the same conditions. RNA precipitation was performed by adding 0.1 vol. of sodium acetate (3 M, pH 5.2) and 2.5 vol. of absolute ethanol to the aqueous phase, before incubation at −20 °C for the night. After centrifugation (16,000 g, 30 min, 4 °C), the pellet was washed with 70% ethanol and dried as described above. The final extract was resuspended in RNase-free water and stored at −80 ° C.

### Quantification of nucleic acids

The quantification of nucleic acids was performed using a NanoDrop™ spectrophotometer (Thermo Scientific™, France). The concentration of DNA and RNA extracts were approximately 60 ng.μL^−1^ and 800 ng.μL^−1^, respectively.

### Nucleic acid sequencing and data treatment

The sequencing of genomic DNA was performed at the Genoscope (Evry, France) from 6 μg of total purified DNA per sample. Librairies were generated with the TruSeq DNA PCR-free protocol, following Illumina instructions. Then, libraries were sequenced on Illumina Hiseq 2500 sequencer as paired-end 150 base reads following Illumina’s instructions, for a total of ~250 M reads per sample. RNA sequencing was performed by GenomEast (IGBMC, Strasbourg) from 3 μg of total purified RNA per sample. The 24 cDNA libraries (8 conditions * 3 biological replicates) were generated using TruSeq Stranded mRNA Sample Preparation Protocol - PN 15031047, version Rev.E Oct 2013, following Illumina’s instructions. Librairies were then sequenced on Illumina Hiseq 4000 sequencer as paired-end 100 base reads following Illumina’s instructions, for a total of 2035 M reads (55–126 M reads per sample). Raw DNAseq reads were aligned on the grapevine reference genome assembly (12X.v2) with gsnap (v.2013) (http://research-pub.gene.com/gmap/). Raw RNAseq reads were aligned on the grapevine reference genome assembly (12X.v2) and its annotation (VCost.v3) (Canaguier et al. 2017) with STAR (v.2.5.3a) (ncbi.nlm.nih.gov/pmc/articles/PMC3530905/). Given that the genomes of the two varieties are very close to the reference genome PN40024, all alignments were filtered in order to keep only the primary alignments, and the alignments with an edit distance >5 were removed. The results of RNAseq alignments for the 24 samples (8 conditions * 3 biological replicates) are presented in the Additional file 6.

### AS analysis and gene expression quantification

For AS analysis, the rMATS software version 4.0.2 (rnaseq-mats.sourceforge.net/) was used to compare replicate biological samples representing two different conditions, either consecutive stages or different varieties. This fast computer program has been conceived to model exon inclusion levels and to evaluate, simultaneously, the individual replicates uncertainty and the variability among replicates, which leads to highly accurate validation of DAS events between the compared conditions. AS events were detected by rMATS using both the RNAseq data BAM files and the grapevine reference genome GTF file. DAS events were statistically validated (FDR ≤ 0.05) for a difference between isoform ratios exceeding the defined threshold of 5% (c). The different types of AS events considered were A3SS, A5SS, ES and IR events. The results were delivered as files summarizing the genomic coordinates of each event, the count of RNAseq reads corresponding to the longest and the shortest isoform, the estimated IL (percent-estimation of the longest isoform among the total) for the two compared conditions, the average ILD between the two conditions, and the results (P and FDR values) of the statistical validation test. We retained only AS events supported by a minimum number of 15 reads (average of three replicates) and an IL value comprised between 0.1 to 0.9 (average of three replicates) in at least one condition. Sequence alignments were visualized with the Integrative Genomics Viewer (IGV 2.8.9) [74] (software.broadinstitute.org/software/igv/) to localize the AS events in the 3′- or 5′-UTRs or in the CDS according to the VCost.v3 annotation, and to compare the genomic sequence of splice sites between the two varieties. Sashimi plots representing the alignment of RNAseq reads to the genome annotation were automatically generated by a dedicated tool of the IGV application. For gene expression quantification, read counts (normalized by the size of the library and the gene length), were generated with featureCounts (v1.5.3) (academic.oup.com/bioinformatics/article/30/7/923/232889) and according to the VCost.v3 annotation. The comparison of gene expression between consecutive developmental stages was then performed with the Sartools R package (v1.7.3) (journals.plos.org/plosone/article?id=10.1371/journal.pone.0157022) and the DESeq2 R package (v1.22.2) (pubmed.ncbi.nlm.nih.gov/25516281/). Differential expression was determined using a log_2_ FC ≥ 0.6 and an FDR cutoff of 0.05.

### Gene ontology analysis

The functional classification of all DAS genes identified in the different comparaisons was carried out using their corresponding v2 annotations and the Gene List Analysis tool of the PANTHER 15.0 Gene Ontology Resource (www.pantherdb.org/), or failing that, by a BLAST search in the genome data resource of the European Bioinformatics Institute (EMBL-EBI) Ensembl Plants (plants.ensembl.org/index.html). The annotation data sets selected in the PANTHER tool were GO Slim- BP, − MF and -CC. GO-term enrichment was statistically determined using the PANTHER overrepresentation test (Fishers’ Exact with the FDR correction, at the threshold of 5%).

## Supplementary Information

**Additional file 1.** Total number of AS events and DAS events in comparisons between consecutive stages or varieties. Retained events were supported by a minimum number of 15 reads of the rarest isoform under at least one condition, and a minimum inclusion level (IL) of 10%.

**Additional file 2.** Description of the 305 unique DAS events identified in stage and variety comparisons.

**Additional file 3.** Functional classification of the genes affected by differential AS in stage and variety comparisons. The classification was performed using the PANTHER GO-slim tool (www.pantherdb.org/). GO terms are split in the three usual categories a Biological Process (BP). b Molecular Function (MF). c Cellular Component (CC). The percent of genes belonging to each category is given on the x-axis.

**Additional file 4.** Differential isoform ratios between Gw and Ri, for fifteen AS events regulated between véraison and mid-ripening in only one variety.

**Additional file 5.** Genes regulated between consecutive stages of berry development at both transcriptional and splicing level. Gene IDs correspond to the VCost.v3 genome annotation.

**Additional file 6.** Number of RNAseq reads and percent of aligned reads for the 24 biological samples (2 varieties * 4 stages * 3 replicates).

## Data Availability

The sequencing data generated and analysed during the current study are available in the European Nucleotide Archive repository, https://www.ebi.ac.uk/ena/browser/view/PRJEB45016. All other data are presented in the manuscript or in the Additional files.

## Abbreviations

A3SS: 3′- splice site
A5SS: 5′- splice site
ABA: Abscisic acid
AS: Alternative splicing
BP: Biological process
CC: Cellular component
CDS: Coding sequence
DAS: Differential AS
ES: Exon skipping
FC: Fold change
GO: Gene ontology
Gw: Gewurztraminer
IL: Inclusion level
ILD: IL difference
IR: Intron retention
MF: Molecular function
NLS: Nuclear localization signal
NMD: Nonsense-mediated mRNA decay
pre-mRNAs: Pre-messenger RNAs
PTC: Premature termination codon
Ri: Riesling
rMATS: Replicate Multivariate-Analysis-of-Transcript-Splicing
SR: Serine-arginine rich
UTR: Untranslated region
