# Supplementary material for: Alternative splicing regulation appears to play a crucial role in grape berry development and is also potentially involved in adaptation responses to the environment
Source: BMC Plant Biol. 2021 Oct 25;21:487. doi: 10.1186/s12870-021-03266-1 (PMC8543832; doi:10.1186/s12870-021-03266-1)
Supplement: Supplementary file 1 — Additional file 1. Total number of AS events and DAS events in comparisons between consecutive stages or varieties. Retained events were supported by a minimum number of 15 reads of the rarest isoform under at least one condition, and a minimum inclusion level (IL) of 10%. [file 12870_2021_3266_MOESM1_ESM.pdf]

Title: Alternative splicing regulation appears to play a crucial role in grape berry development and is also potentially involved in adaptation responses to the environment

Journal: BMC Plant Biology

Authors: Pascale Maillot, Amandine Velt, Camille Rustenholz, Gisèle Butterlin, Didier Merdinoglu, Eric Duchêne

Corresponding author: Pascale Maillot, SVQV, INRAE - University of Strasbourg, 68000 Colmar, France, France, pascale.maillot@inrae.fr

		Comparisons between consecutive stages[1]						Comparisons between Gw and Ri at each stage[1]			
		S1 vs S2		S2 vs S3		S3 vs S4					
		Gw	Ri	Gw	Ri	Gw	Ri	S1	S2	S3	S4
A3SS	AS events	398	352	394	300	403	324	375	368	346	371
	DAS events	*0*	*0*	*2*	*1*	*3*	*21*	*32*	*36*	*33*	*19*
A5SS	AS events	178	133	161	120	162	125	162	151	140	146
	DAS events	*0*	*0*	*0*	*0*	*4*	*12*	*15*	*18*	*19*	*9*
ES	AS events	409	307	412	300	502	401	377	386	377	492
	DAS events	*0*	*2*	*3*	*5*	*33*	*52*	*38*	*46*	*52*	*40*
IR	AS events	162	121	154	107	165	122	155	147	135	157
	DAS events	*1*	*0*	*2*	*2*	*20*	*23*	*18*	*20*	*20*	*19*

[1] S1: green berry at 6 weeks post-flowering, S2: hard berry at mid-véraison, S3: soft berry at mid-véraison, S4: mid-ripening

Additional file 1. Total number of AS events and DAS events in comparisons between consecutive stages or varieties. Retained events were supported by a minimum number of 15 reads of the rarest isoform under at least one condition, and a minimum inclusion level (IL) of 10 %
