# Supplementary material for: Alternative splicing regulation appears to play a crucial role in grape berry development and is also potentially involved in adaptation responses to the environment
Source: BMC Plant Biol. 2021 Oct 25;21:487. doi: 10.1186/s12870-021-03266-1 (PMC8543832; doi:10.1186/s12870-021-03266-1)
Supplement: Supplementary file 3 — Additional file 3. Functional classification of the genes affected by differential AS in stage and variety comparisons. The classification was performed using the PANTHER GO-slim tool (www.pantherdb.org/). GO terms are split in the three usual categories a Biological Process (BP). b Molecular Function (MF). c Cellular Component (CC). The percent of genes belonging to each category is given on the x-axis. [file 12870_2021_3266_MOESM3_ESM.pdf]

Title: Alternative splicing regulation appears to play a crucial role in grape berry development and is also potentially involved in adaptation responses to the environment

Journal: BMC Plant Biology

Authors: Pascale Maillot, Amandine Velt, Camille Rustenholz, Gisèle Butterlin, Didier Merdinoglu, Eric Duchêne

Corresponding author: Pascale Maillot, SVQV, INRAE - University of Strasbourg, 68000 Colmar, France, France, pascale.maillot@inrae.fr

A.

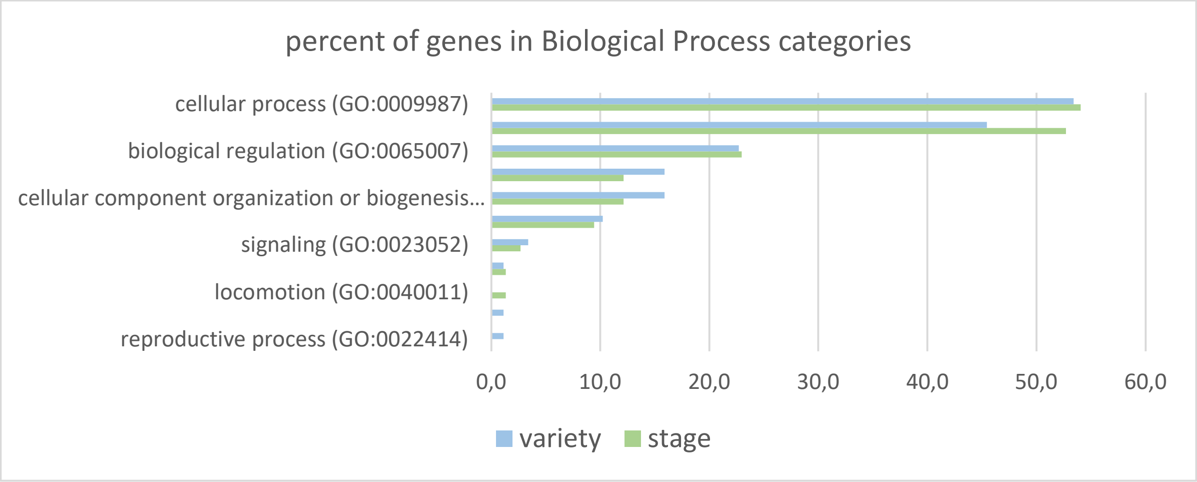

B.

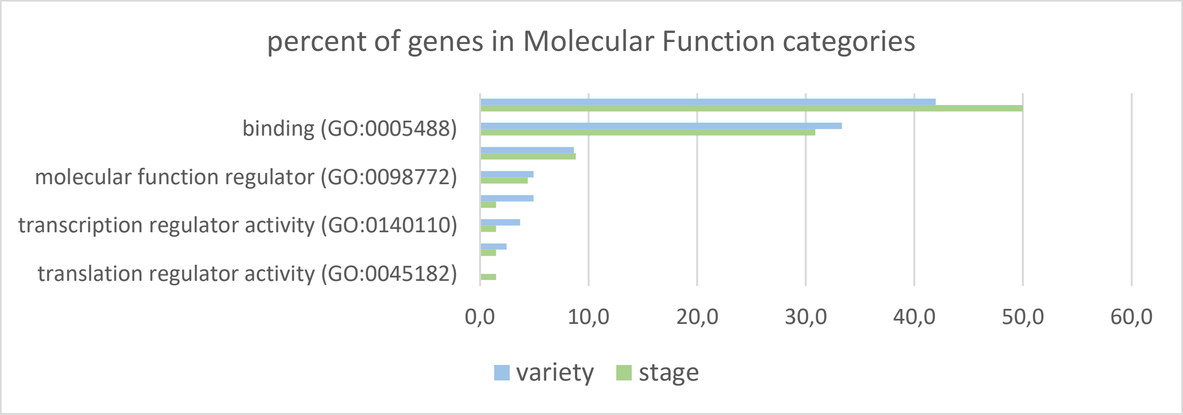

C.

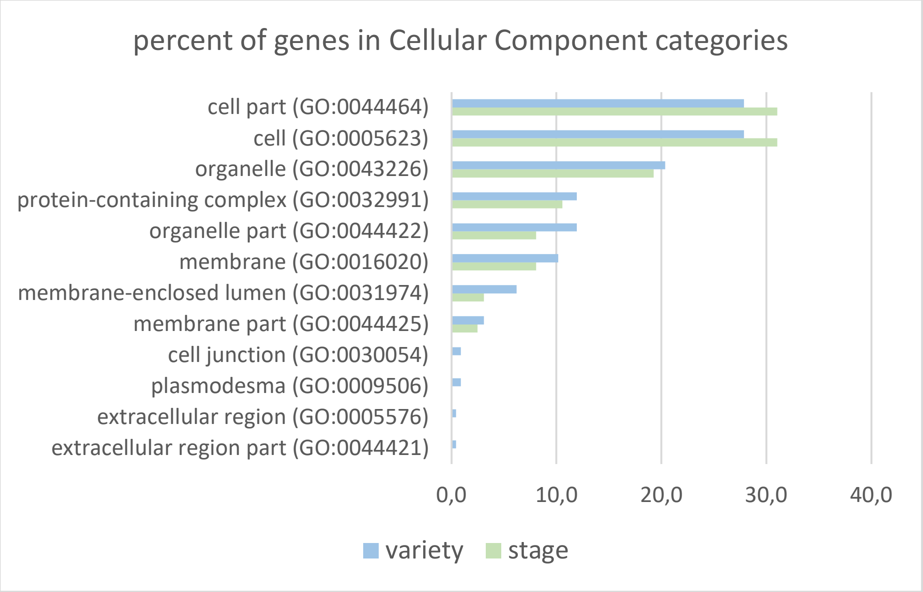

Additional file 3. Functional classification of the genes affected by differential AS in stage and variety comparisons. The classification was performed using the PANTHER GO-slim tool (www.pantherdb.org/). GO terms are split in the three usual categories **a** Biological Process (BP). **b** Molecular Function (MF). **c** Cellular Component (CC). The percent of genes belonging to each category is given on the x-axis
