# Supplementary material for: Alternative splicing regulation appears to play a crucial role in grape berry development and is also potentially involved in adaptation responses to the environment
Source: BMC Plant Biol. 2021 Oct 25;21:487. doi: 10.1186/s12870-021-03266-1 (PMC8543832; doi:10.1186/s12870-021-03266-1)
Supplement: Supplementary file 4 — Additional file 4. Differential isoform ratios between Gw and Ri, for fifteen AS events regulated between véraison and mid-ripening in only one variety. [file 12870_2021_3266_MOESM4_ESM.pdf]

Title: Alternative splicing regulation appears to play a crucial role in grape berry development and is also potentially involved in adaptation responses to the environment

Journal: BMC Plant Biology

Authors: Pascale Maillot, Amandine Velt, Camille Rustenholz, Gisèle Butterlin, Didier Merdinoglu, Eric Duchêne

Corresponding author: Pascale Maillot, SVQV, INRAE - University of Strasbourg, 68000 Colmar, France, France, pascale.maillot@inrae.fr

			ILD[1] – Gw vs Ri at the different stages[2]			
**Gene ID**	**AS** (region)	**specificity**	**S1** (FDR)	**S2** (FDR)	**S3** (FDR)	**S4** (FDR)
Ri-specific or -preferential events						
Vitvi01g00819	**IR** (CDS)	ILs-Gw = 1	**0.55** (0)	**0.66** (0)	**0.53** (0)	not different[3]
Vitvi13g01915	**A5SS** (CDS)	ILs-Gw = 1	**0.36** (0)	**0.37** (0)	**0.35** (0)	not different[3]
Vitvi14g02541	**ES** (CDS)	ILs-Gw = 1	**0.52** (0)	**0.43** (1.8 E-13)	**0.40** (0)	not different[3]
Vitvi15g00621	**IR** (3'UTR)	ILs-Gw ≤ 0.02	**-0.99** (0)	**-0.97** (0)	**-0.98** (0)	**-0.81** (0) [3]
Vitvi17g00770	**A3SS** (CDS)	ILs-Gw ≤ 0.02	**-0.54** (0)	**-0.60** (0)	**-0.55** (0)	not different[3]
Other events						
Vitvi01g02098	**A3SS** (3'UTR)	_	**-0.13** (3.5 E-05)	**-0.13** (2.1 E-05)	**-0.15** (7.0 E-09)	not different[3]
Vitvi02g00597	**A5SS** (CDS)	_	**0.11** (ns)	**0.11**(ns)	**0.16** (1.6 E-04)	**-0.17** (3.1 E-06) [3]
Vitvi06g01662	**A3SS** (5'UTR)	_	**0.59** (0)	**0.54** (0)	**0.62** (0)	not different[3]
Vitvi13g02138	**ES** (5'UTR)	_	**-0.33** (1.6 E-03)	**-0.30** (2.6 E-03)	**-0.23** (3.5 E-03)	not different[4]
Vitvi14g00256	**ES** (CDS)	_	**-0.59** (0)	**-0.60** (0)	**-0.55** (0)	not different[3]
Vitvi15g01145	**A5SS** (5'UTR)	_	weak expression	**-0.23** (9.0 E-08)	**-0.17** (3.6 E-05)	not different[4]
Vitvi15g01145	**A3SS** (5'UTR)	_	weak expression	**-0.19** (4.2 E-04)	**-0.15** (1.3 E-03)	not different[4]
Vitvi15g01145	**IR** (5'UTR)	_	weak expression	**-0.14** (1.4 E-05)	**-0.09** (1.8 E-02)	not different[4]
Vitvi15g01173	**ES** (CDS)	_	**-0.24** (1.1 E-05)	**-0.22** (1.1 E-03)	**-0.19** (5.4 E-05)	not different[4]
Vitvi17g00038	**A3SS** (5'UTR)	_	**-0.24** (0)	**-0.28** (0)	**-0.26** (0)	not different[3]

[1] ILD values are means of three biological replicates, significant at FDR ≤ 0.05

[2] S1: green berry at 6 weeks post-flowering, S2: hard berry at mid-véraison, S3: soft berry at mid-véraison, S4: mid-ripening

[3] AS event regulated between S3 and S4 in Ri

[4] AS event regulated between S3 and S4 in Gw

Additional file 4. Differential isoform ratios between Gw and Ri, for fifteen AS events regulated between véraison and mid-ripening in only one variety
