# Supplementary material for: Alternative splicing regulation appears to play a crucial role in grape berry development and is also potentially involved in adaptation responses to the environment
Source: BMC Plant Biol. 2021 Oct 25;21:487. doi: 10.1186/s12870-021-03266-1 (PMC8543832; doi:10.1186/s12870-021-03266-1)
Supplement: Supplementary file 5 — Additional file 5. Genes regulated between consecutive stages of berry development at both transcriptional and splicing level. Gene IDs correspond to the VCost.v3 genome annotation. [file 12870_2021_3266_MOESM5_ESM.pdf]

Title: Alternative splicing regulation appears to play a crucial role in grape berry development and is also potentially involved in adaptation responses to the environment

Journal: BMC Plant Biology

Authors: Pascale Maillot, Amandine Velt, Camille Rustenholz, Gisèle Butterlin, Didier Merdinoglu, Eric Duchêne

Corresponding author: Pascale Maillot, SVQV, INRAE - University of Strasbourg, 68000 Colmar, France, France, pascale.maillot@inrae.fr

	Comparisons between consecutive stages[1]											
	S1 vs S2				S2 vs S3				S3 vs S4			
	Gw		Ri		Gw		Ri		Gw		Ri	
Gene ID	Transcr Reg $\log_2$ FC (FDR)[2]	Splicing Reg[3]	Transcr Reg $\log_2$ FC (FDR)[2]	Splicing Reg[3]	Transcr Reg $\log_2$ FC (FDR)[2]	Splicing Reg[3]	Transcr Reg $\log_2$ FC (FDR)[2]	Splicing Reg[3]	Transcr Reg $\log_2$ FC (FDR)[2]	Splicing Reg[3]	Transcr Reg $\log_2$ FC (FDR)[2]	Splicing Reg[3]
Vitvi03g00047	_	_	_	_	_	_	_	_	**1.1** (2.8 E-03)	yes	_	_
Vitvi03g01012	_	_	_	_	**1.0** (2.3 E-23)	_	_	_	_	yes	_	_
Vitvi04g00040	_	_	_	_	**1.9** (1.5 E-16)	_	**1.3** (2.9 E-08)	_	_	yes	_	_
Vitvi04g00388	_	_	_	_	**1.0** (1.1 E-02)	_	_	_	**1.6** (1.3 E-05)	yes	**1.4** (1.5 E-04)	yes
Vitvi04g01198	_	yes	_	_	_	yes	**-1.1** (2.2 E-05)	_	**-1.2** (1.6 E-06)	_	_	_
Vitvi05g00214	**-1.7** (4.9 E-11)	_	**-1.0** (1.6 E-04)	_	_	_	_	_	_	_	**-1.5** (1.5 E-10)	yes
Vitvi05g00364	_	_	_	_	_	_	_	_	_	_	**-1.1** (1.2 E-11)	Yes
Vitvi05g00462	_	_	_	_	_	_	_	_	_	_	**2.0** (7.0 E-16)	Yes
Vitvi05g00530	_	_	_	_	_	_	_	_	**-1.0** (1.5 E-06)	_	**-1.1** (1.4 E-07)	Yes
Vitvi06g00213	_	_	_	_	_	_	_	_	_	_	**1.4** (5.7 E-06)	Yes
Vitvi07g00544	_	_	_	_	_	_	_	_	**1.1** (3.6 E-03)	yes	_	_
Vitvi07g01473	_	_	_	_	_	_	_	_	**1.1** (3.2 E-03)	yes	**1.4** (2.2 E-04)	_
Vitvi08g00972	_	_	_	_	**2.3** (5.3 E-35)	_	**1.3** (1.2 E-11)	_	_	yes	_	_
Vitvi11g01400	_	_	**1.0** (1.1 E-06)	_	**1.4** (8.2 E-13)	_	**1.7** (9.5 E-19)	_	_	_	_	Yes
Vitvi13g01687	_	_	_	_	_	_	_	_	_	yes	**1.4** (1.4 E-04)	_
Vitvi14g00256	_	_	_	_	_	_	_	_	_	_	**1.2** (6 .1 E-26)	Yes
Vitvi14g01906	_	_	_	_	_	_	_	_	**1.3** (2.0 E-06)	_	_	Yes
Vitvi15g01191	_	_	_	_	_	_	_	_	**-1.3** (2.5 E-30)	_	**-1.2** (9.7 E-24)	Yes
Vitvi17g00561	_	_	_	_	**1.0** (9.2 E-10)	_	_	_	_	yes	_	Yes
Vitvi17g00770	_	_	_	_	_	_	_	_	**-1.3** (1.5 E-11)	_	_	Yes
Vitvi18g01553	_	_	_	_	_	_	_	_	**1.3** (3.8 E-15)	_	_	Yes

Vitvi19g00041	_	_	**1.6** (8.1 E-30)	_	_	_	**1.0** (1.8 E-13)	_	_	_	_	**Yes**
Vitvi19g00604	_	_	_	_	_	_	_	_	**1.6** (3.9 E-21)	_	_	**yes**
Vitvi19g01705	_	_	_	_	**-1.4** (4.1 E-05)	_	**-1.0** (4.0 E-03)	_	_	**yes**	_	_
Vitvi19g01964	_	_	**1.2** (5.0 E-05)	_	**1.7** (2.1 E-08)	_	**1.6** (1.7 E-08)	**yes**	**1.4** (1.7 E-06)	_	_	_

[1] S1: green berry at 6 weeks post-flowering, S2: hard berry at mid-véraison, S3: soft berry at mid-véraison, S4: mid-ripening

[2] Transc Reg: regulation at the transcriptional level, validated for a $\log_2$ fold change (FC) ≥ 1.0, significant at FDR ≤ 0.05

[3] Splicing Reg: regulation at the splicing level, as reported in Online Resource 1

Additional file 5. Genes regulated between consecutive stages of berry development at both transcriptional and splicing level. Gene IDs correspond to the VCost.v3 genome annotation
